# Difference between cerebral embolic events following Transcatheter Aortic Valve Implantation (TAVI) and Surgical Aortic Valve Replacement (SAVR): a diffusion weighted MRI study

**DOI:** 10.1186/1532-429X-15-S1-O59

**Published:** 2013-01-30

**Authors:** Akhlaque Uddin, Timothy Fairbairn, Ibrahim Djoukhader, Stuart Currie, Christopher D Steadman, Manish Motwani, Ananth Kidambi, Anthony Goddard, Daniel Blackman, Gerry P McCann, Sven Plein, John P Greenwood

**Affiliations:** 1 Academic Unit of Cardiovascular Medicine, Multidisciplinary Cardiovascular Research Centre (MCRC) & Leeds Institute of Genetics, Health and Therapeutics, Leeds, UK; 2 Department of Neuroradiology, Leeds Teaching Hospitals NHS trust, Leeds General Infirmary, Leeds, UK; 3 National Institute for Health Research(NIHR) Leicester Cardiovascular Biomedical Research Unit, University of Leicester, Leicester, UK; 4 Department of Cardiology, Leeds Teaching Hospitals NHS trust, Leeds General Infirmary, Leeds, UK

## Background

Transcatheter Aortic Valve Implantation (TAVI) is used to treat symptomatic severe aortic stenosis in a non-surgical high risk population. The incidence of stroke and micro-infarction is higher in the TAVI population compared to surgical aortic valve replacement (SAVR) at 30 days, which may be due to various factors such as valve calcification and aortic atheroma. However, the natural history and clinical consequences of micro-infarction is unknown.

## Methods

Cerebral imaging was conducted before TAVI/SAVR, at <7 days post-procedure and 6 months post-procedure. MRI scans were performed on a 1.5T system (Intera, Philips or Avanto, Siemens) using a protocol of T2 weighted fast field echo, T2 turbo field echo and diffusion weighted imaging (DWI) (22 slices, 5 mm thick, 1mm gap, FOV 350, RFOV 100). Three neuroradiologists independently analysed the scans and were blinded to clinical details to avoid bias. New cerebral lesions were measured (<5 mm or>5 mm) and the vascular territory described. Quantification of cerebral infarct lesion volume was performed by manual planimetry using post processing software (Qmass 7.2 Medis, The Netherlands).

## Results

**Figure 1:**
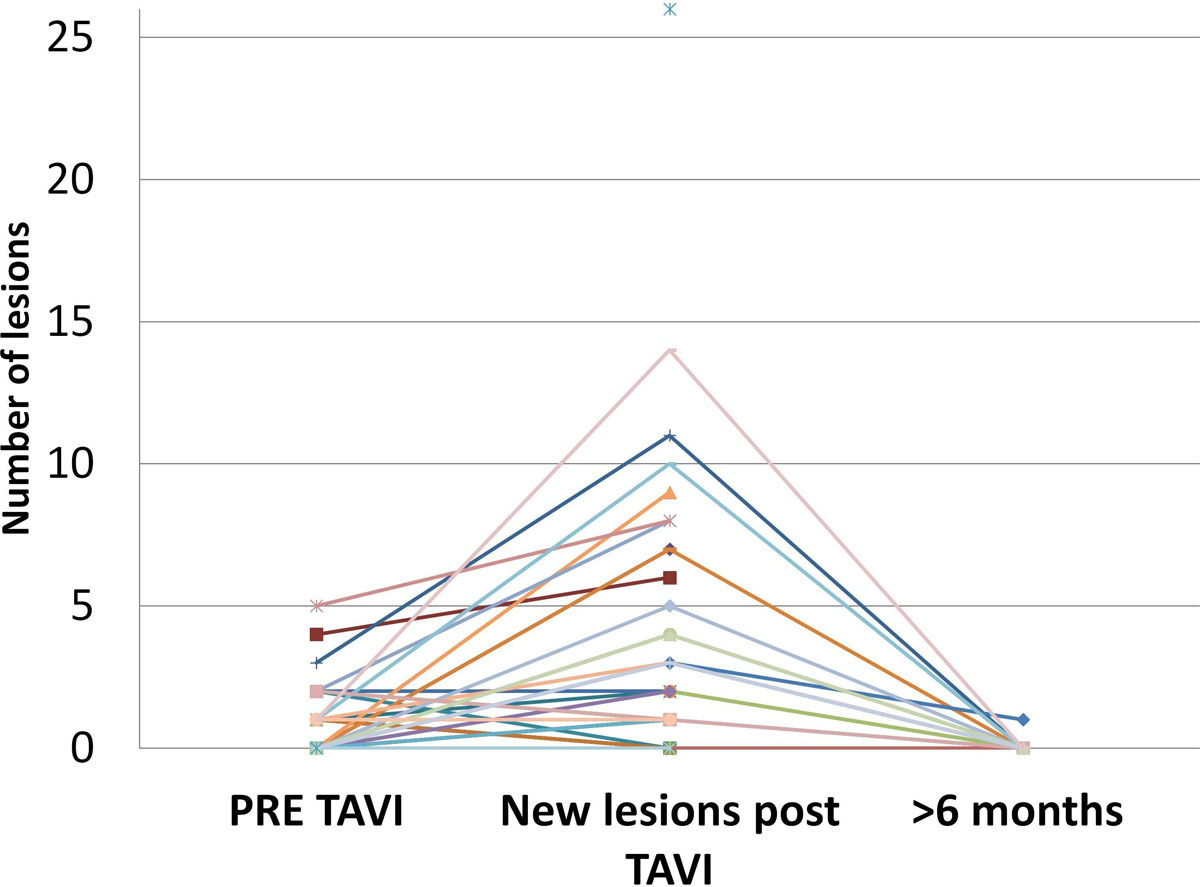
Number of lesions on brain diffusion weighted imaging (DWI) before and after TAVI in 45 patients and at 6 months in 17 patients.

45 TAVI and 21 SAVR patients were studied. Mean age for TAVI was 80±5.9 years, for SAVR 69±8.8 years: Logistic EuroSCORE for TAVI was 19.1±13, for SAVR 7.1±2.7. In the TAVI patients 82% had new embolic lesions on DWI (Figure 1) compared to 47.6% of the SAVR group. Two TAVI patients had a clinical stroke with none in the SAVR group. There was a higher cerebral infract lesion volume in TAVI group compared to SAVR group (1.74±2.8ml vs. 0.41±0.48ml p=0.01). At 6 months 17 TAVI patients (11 were unable to attend, 10 had died, 7 had a pacemaker) and 18 SAVR (1 died and 1 was unable to attend) were re-scanned. Only 1 TAVI and 1 SAVR patient had a new subclinical micro-infarct. All previously detected micro-infarcts had completely resolved.

**Figure 2:**
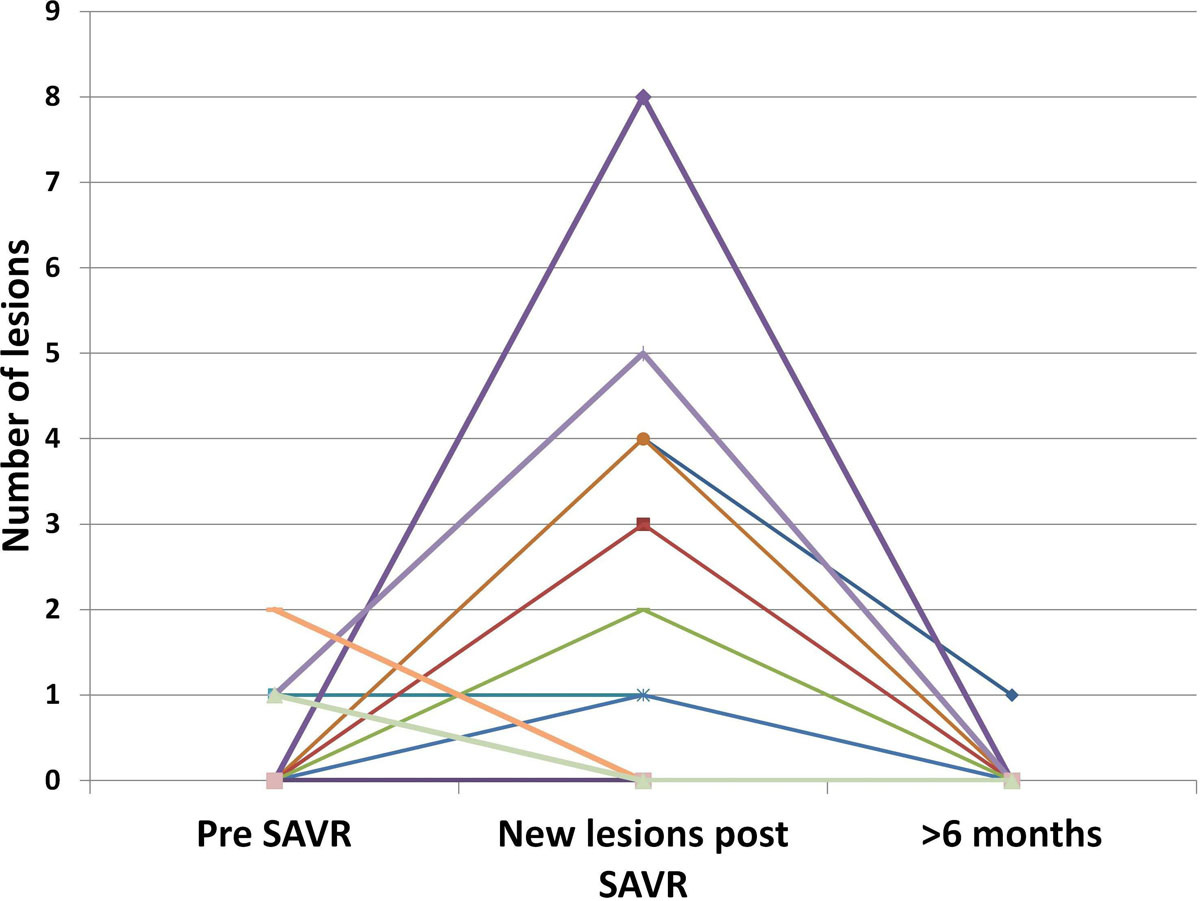
Number of lesions on brain diffusion weighted imaging(DWI) before and after surgical aortic valve replacement(SAVR) in 21 patients and at 6 months in 18 patients

## Conclusions

There is a significantly greater number of new micro-embolic events after TAVI compared to SAVR. However all of these lesions appear to completely resolve by 6 months.

## Funding

SP is funded by a British Heart Foundation fellowship (FS/10/62/28409).

SP and JPG receive an educational research grant from Philips Healthcare.

